# A Global Population Genetic Study of Pantala flavescens

**DOI:** 10.1371/journal.pone.0148949

**Published:** 2016-03-02

**Authors:** Daniel Troast, Frank Suhling, Hiroshi Jinguji, Göran Sahlén, Jessica Ware

**Affiliations:** 1 Department of Biology, Rutgers University, Newark, New Jersey, United States of America; 2 Institut für Geoökologie, Technische Universität Braunschweig, Braunschweig, Germany; 3 School of Food, Agricultural and Environmental Sciences, Miyagi University, Miyagi, Japan; 4 Ecology and Environmental Science, Halmstad University, Halmstad, Sweden; National Cheng-Kung University, TAIWAN

## Abstract

Among terrestrial arthropods, the dragonfly species *Pantala flavescens* is remarkable due to their nearly global distribution and extensive migratory ranges; the largest of any known insect. Capable of migrating across oceans, the potential for high rates of gene flow among geographically distant populations is significant. It has been hypothesized that *P*. *flavescens* may be a global panmictic population but no sufficient genetic evidence has been collected thus far. Through a population genetic analysis of *P*. *flavescens* samples from North America, South America, and Asia, the current study aimed to examine the extent at which gene flow is occurring on a global scale and discusses the implications of the genetic patterns we uncovered on population structure and genetic diversity of the species. This was accomplished using PCR-amplified cytochrome oxidase one (CO1) mitochondrial DNA data to reconstruct phylogenetic trees, a haplotype network, and perform molecular variance analyses. Our results suggested high rates of gene flow are occurring among all included geographic regions; providing the first significant evidence that *Pantala flavescens* should be considered a global panmictic population.

## Introduction

Populations vary greatly in size, range, and the movement of its individuals. The scale and timing of species movements, or migrations, are a key component in determining how populations are structured and the degree to which that structure is maintained [1]. Ecological, genetic, and evolutionary implications can be tied to the dispersal-timing and -capabilities of a species with the most profound impacts occurring at a population level [1]. The rate at which individuals are exchanged among geographically distant populations, linked through dispersal, is perhaps the most important factor when it comes to examining the overall impact that migrations have on a population level. Panmixia occurs when, due to large or connected ranges, and/or high dispersal capabilities, populations are effectively one large grouping of individuals, with random mating occurring freely; this has been found in, for example, moose [2,3,4].

Animals with exceptionally large ranges, such as whales and birds, may be the most commonly thought of when it comes to long distance migrations, yet there are many insects which are just as capable of traversing incredible distances. Few insects, however, are as capable of long distance travel as dragonflies. The order Odonata, encompassing dragonflies (suborder Anisoptera) and damselflies (suborder Zygoptera), comprises taxa such as the dragonfly species *Anax junius* (Aeshnidae) and *Pantala flavescens* (Libellulidae), which have been suggested to migrate long distances.

With a nearly global distribution, *Pantala flavescens*, commonly known as the “wandering glider” or the “globe skimmer”, may be the most widespread of any known dragonfly species [5,6]. Primarily circumtropical in their distribution [7], they can also be found in many temperate areas including the northeastern United States and southern Canada [8] as well as northeastern China [9]. Although there are no recorded breeding populations in Europe [8], or much of the far northern hemisphere, there are still instances where *P*. *flavescens* has been found far outside its normal range; these include sightings as far north as the Baltic sea [10] as well as remote island locations in Micronesia [11]. Although not rare, as *P*. *flavescens* is one of the most common dragonfly species on the planet, it is still of interest to academics and the public alike, due in part to its remarkable dispersal abilities.

The extensive range of *P*. *flavescens* provides important insight into what may be considered the true defining characteristic of this species: their migratory behavior. The migration of dragonfly species is well documented and occurs on all continents with the exception of Antarctica [12]. Yet, of the approximately 6,000 known Odonata species in existence [13], it is estimated that as few as 25–50 of these species are migratory [5]. Even among such limited company, the distance and scale of *P*. *flavescens* migrations is unusually broad. With an enlarged hindwing base to aid in gliding [8,14,15], allowing them to travel extraordinary distances, *P*. *flavescens* has the longest known migration not just of any dragonfly, but of any known insect [6,14,16]. While many are familiar with the migration of the monarch butterfly in North America, which can travel an incredible distance of up to 4,000km in each direction during their multigenerational migration to Mexico and back [16,17], *P*. *flavescens* has a migration route which can more than double the overall length of the Monarchs' migration. During their documented multigenerational migration route from India to east Africa and back again, swarms of millions of *P*. *flavescens* can cover a total distance ranging from, or possibly exceeding, 14,000–18,000km [5,14].

The sheer scope of this migration is remarkable, but it is also the only known transoceanic migration by an insect; *P*. *flavescens* flies more than 3,500km over open waters across the Indian Ocean [7,14,15]. This truly unique behavior is a stark contrast to other migratory dragonflies, such as *Anax junius*, which actively avoid flying over open waters [5,15]. Expansive oceanic crossings may be considered commonplace for animals such as sea turtles, marine fish, birds, and whales [18,19,20], yet *P*. *flavescens* has proven to be equally capable despite their considerably smaller stature.

For many migratory animals, successful completion of their life cycle is dependent upon timing their migrations in accordance with seasonal and temporal patterns in order to maximize their time in habitats more conducive to successful breeding. Requiring freshwater for reproduction, the migrations of *P*. *flavescens* across bodies of salt water seem like a counterintuitive, overly risky life strategy, but the risks associated with this behavior are not without their rewards. It has been shown that *P*. *flavescens* embarks on these migrations following shifting weather fronts at different times of the year to take advantage of seasonal rainfall, exploiting ephemeral, freshwater rain pools in which they reproduce [14]. Their larvae have a remarkably short development time which can be as rapid as 38–65 days [21,22], allowing them to mature before the temporary pools, in which they develop, dry out. Newly emerged adults then continue along these migratory routes, following seasonal rainfall patterns and reproducing along the way as they complete their leg of the migratory circuit.

Shifting fronts, such as the Intertropical Convergence Zone (ITCZ), not only provide essential freshwater pools for breeding, but the associated winds are what allow *P*. *flavescens* to migrate long distances while flying at altitudes of over 1,000m [9,14,23]. Physical and behavioral adaptations such as the ability to compensate for wind drift [9,15], slope soaring behavior [24], feeding on aerial plankton and other small insects [15,25], and an enlarged hind wing base [8,14,15], all contribute to the process of energy conservation; critical for long distance migratory flights. Despite these adaptations, *P*. *flavescens* is still largely at the mercy of the winds upon which they rely, resulting in them being blown far off their intended course in some instances. Populations have been documented in locations as remote as Easter Island where a unique population has exhibited both morphological and behavioral adaptations towards being non-migratory (e.g., they crouch low against the substrate rather than lift their tarsae when wind passes over their wings and possess smaller hindwings than continental populations) [7].

**Fig 1:**
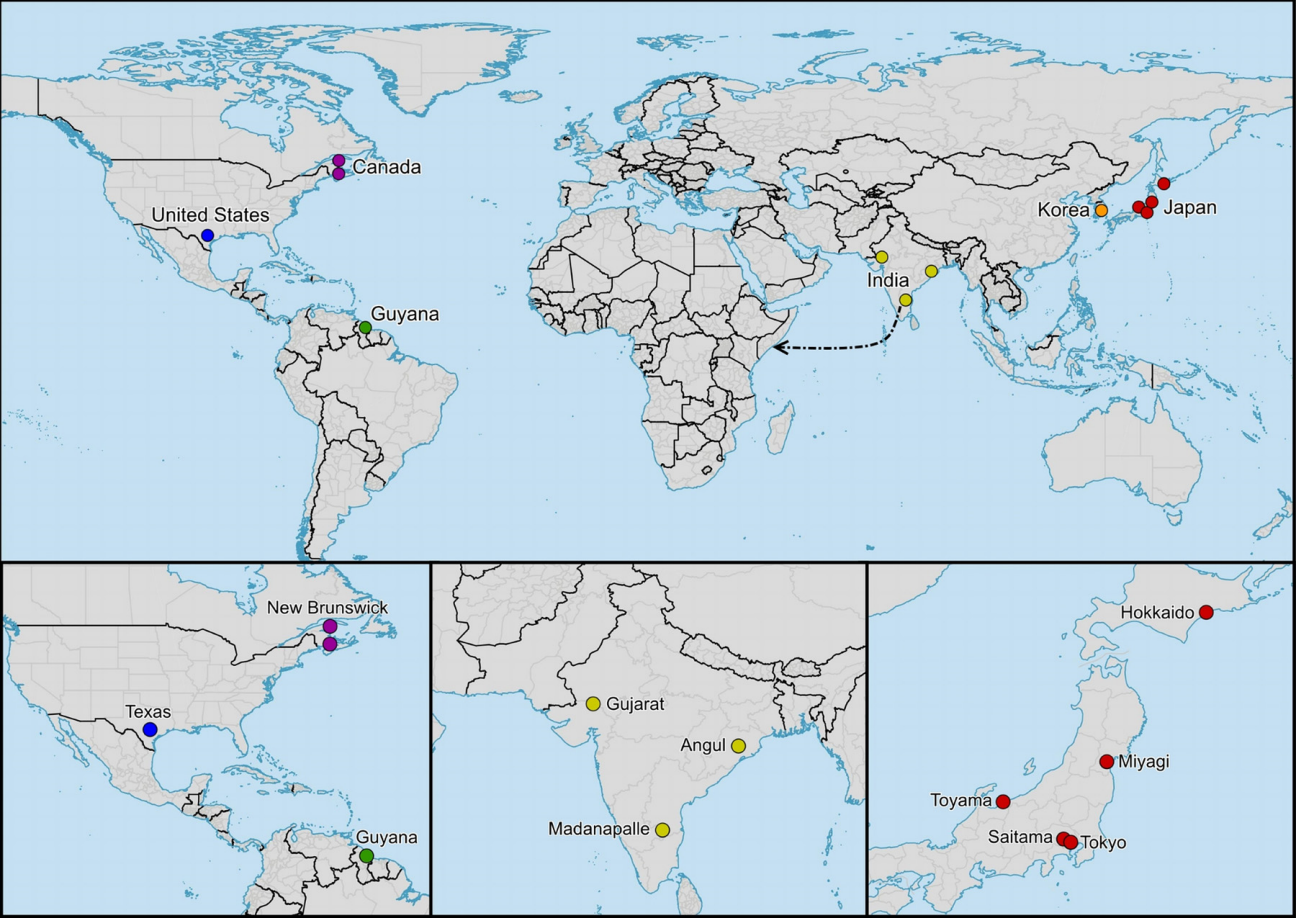
Distribution of Individuals. Distribution of *Pantala flavescens* individuals used in this study. The dotted line represents the suggested India to Africa migration route.

The relationship between the distribution range of a species and the migratory range of a single individual of that species can result in multiple, geographically isolated populations or, in some cases, a single, interbreeding population on both small and large scales [18]. Migratory behavior results in populations that would otherwise be geographically isolated to be linked via the exchange of individuals and genes among multiple populations, impacting the population structure of that species. The geographic distribution and migratory behavior of *P*. *flavescens* present a unique opportunity to ask questions regarding the amount of gene flow that may be occurring on a global scale as well as its influence on both the population structure and genetic diversity of the species. If a significant amount of gene flow is occurring across continents it is possible that *P*. *flavescens* could be considered a global, panmictic population, a phenomenon that is rare among animals (occurring, for example, in sea coral [26], European Eel [27,28], and some fish [29,30]). In this study we aim to address these questions through a population genetic analysis using mitochondrial DNA data of geographically distinct populations of *P*. *flavescens* from North America, South America, and Asia (Fig 1).

## Materials and Methods

### Sampling

Samples of *Pantala flavescens* from Guyana, Japan, India, and the United States were acquired for DNA extraction and sequencing from the field and museum collections. Guyana samples were collected on private land with permission of the land owners, and field permits were issued by the Guyana Environmental Protection Agency. Field studies in Japan, India, and the United States were carried out on private lands with permission from the land owners. Field samples were numbered sequentially by country as follows: Guyana 1–21, Japan 1–15, India 1–2, U.S.A. 1–2. No protected or endangered species were involved in any field studies. All samples that were sequenced for use in this study are deposited in the Ware lab private collection at Rutgers University, Newark.

Samples from Japan, provided by Hiroshi Jinguji, were collected from Saitama, Miyagi, and Tokyo prefectures between July 2013 and July 2014. All Japanese samples were stored individually in ethanol. Samples from Guyana and the United States were collected by various members of Jessica Ware's lab, treated with acetone, then dried, and stored individually. Guyana samples were collected from the Demerara-Mahaica region in North East Guyana over a period of dates ranging from July 2011 through August 2013. United States samples were collected in Comal County, Texas in August 2013. India samples were collected from the Angul district of Odisha and from Madanapalle in the Chittoor district of Andhra Pradesh in August 2012. India samples were collected by an Indian citizen and sequenced for use by the Ware lab. Additional sequences used in this analysis from India, Korea, Japan, and Canada were sourced from NCBI GenBank and BOLD Systems; *P*. *flavescens* is an easily identified species, and thus the risk of GenBank and BOLD Systems samples being misidentified is low. Including the sequences sourced from NCBI GenBank and BOLD Systems a total of 49 *P*. *flavescens* CO1 sequences were analyzed: 21 from Guyana, 17 from Japan, 5 from India, 2 from Korea, 2 from Canada, and 2 from the United States.

### DNA Extraction and PCR Amplification

DNA extraction was performed on leg and thorax tissue of the *P*. *flavescens* samples using a Qiagen DNeasy Blood and Tissue Kit. The mitochondrial gene cytochrome oxidase one (CO1) was chosen to amplify the extracted samples; this was done using two sets of CO1 primers as listed in Table 1. PCR amplification was performed in 25μl reactions with each reaction consisting of 12.5μl of Taq 2x master mix solution, 1μl of each primer (forward and reverse, both diluted to 1x concentration), 5μl of DNA template, and 5.5μl of RNase-free water. Two thermal cycler programs were used, one for each set of primers. Successfully amplified samples were both purified and sequenced by Macrogen (NYC, NY, USA) for forward and reverse primer sequences. A total of 38 samples were sequenced: 21 from Guyana (samples labeled Guyana 1–21), 2 from the United States (samples labeled U.S.A. 1–2), and 15 from Japan (samples labeled Japan 1–15). All sequences were deposited in NCBI GenBank (accession numbers KU641567-KU641604).

**Table 1:** CO1 Primers.

CO1 Primers	Sequence	Tm °C
LCO (forward)	5' GGTCAACAAATCATAAAGATATTGG 3'	58.0°
HCO (reverse)	5' TAAACTTCAGGGTGACCAAAAAATCA 3'	59.9°
coi1709 (forward)	5' TAATTGGAGGATTTGGAAATTG 3'	55.2°
coi2191 (reverse)	5' CCYGGTARAATTARAATRTARACTTC 3'	59.1°

### Sequence Analysis

For each sample, forward and reverse sequences were assembled into a consensus sequence, or contig, using Geneious software version 8.1.2 [31]. These sequences were then combined with all available *P*. *flavescens* CO1 gene sequences from both NCBI GenBank and BOLD Systems for analysis. All sequences were initially aligned using ClustalX 2.1 [32], followed by manual alignment in Mesquite v.3.02 [33]. *Trithemis festiva* was chosen as the outgroup based on Ware et al. [34] and Pilgrim and von Dohlen [35].

**Fig 2:**
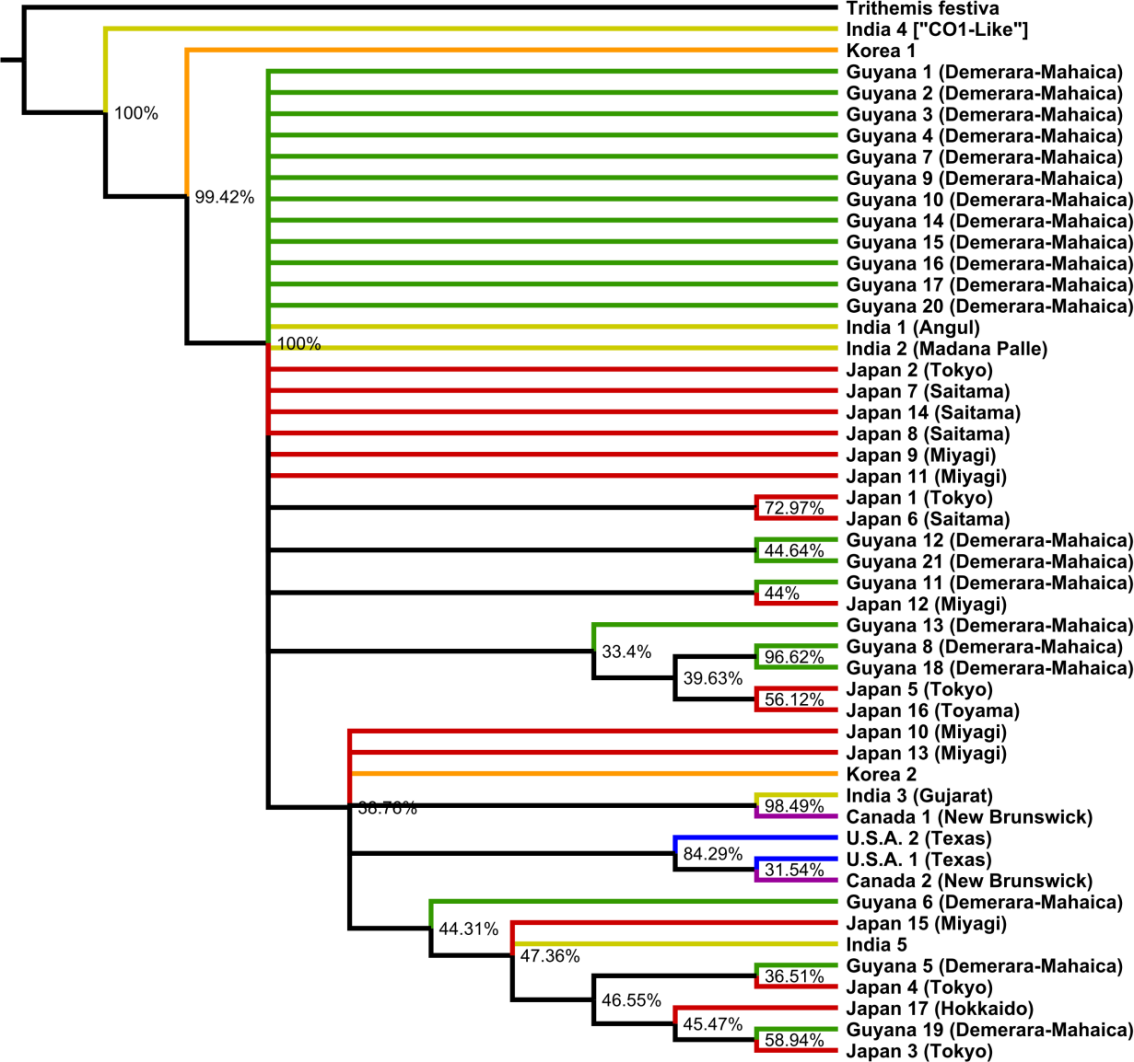
MrBayes Bayesian majority rule consensus tree. Tree based on CO1. Known locations of samples are indicated in parentheses.

**Fig 3:**
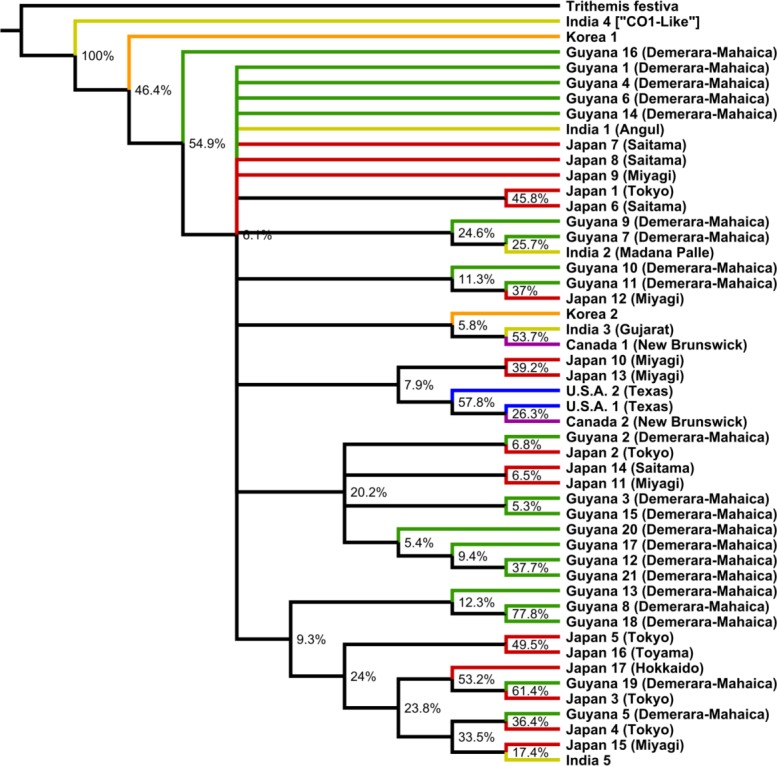
GARLI majority rule consensus tree. Tree based on CO1. Known locations of samples are indicated in parentheses.

As determined by jModelTest 2.1.6 [36], the best nucleotide substitution model was found to be TPM2uf+I+G. We thus implemented the GTR+I+G model, which is the closest model to TPM2uf that can be implemented in Mr. Bayes. The parameters of this model were used for both Bayesian and maximum likelihood analysis. Bayesian analysis was performed using MrBayes v3.2.3 x64 [37,38] running on CIPRES [39] to determine a posterior probability distribution. Two Markov chain Monte Carlo (MCMC) runs were performed; each run consisted of 4 chains running over the course of 10,000,000 generations with sampling occurring every 1,000 generations. Run convergence and a 10% burn-in value were confirmed using Tracer 1.6 [40]. Likelihood analysis was carried out using GARLI 2.01 [41] to reconstruct both a best likelihood tree (Fig 2) and a 50% majority rule, 1,000 repetition bootstrap consensus tree (Fig 3). These trees were summarized and posterior probability support was applied using SumTrees 3.3.0 distributed through the DendroPy 3.12.0 package [42]. Both trees were visualized using FigTree v1.4.2 (available at http://tree.bio.ed.ac.uk/software/figtree/).

**Fig 4:**
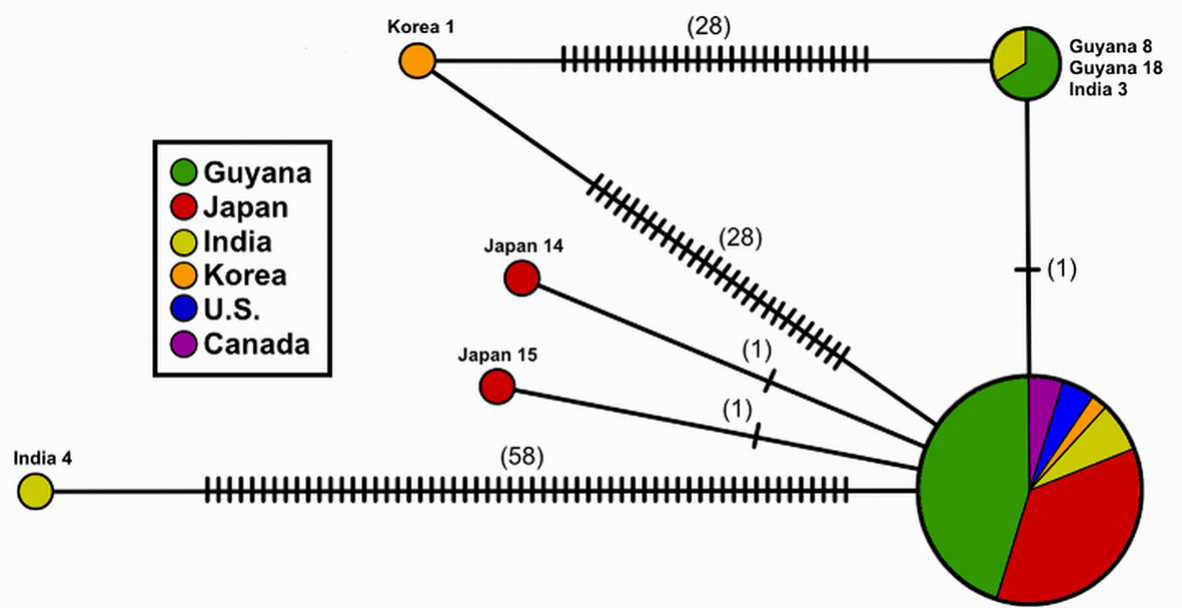
Minimum Spanning Haplotype Network. All haplotypes are based on the CO1 gene except for “India 4” which is “CO1-like”. Numbers in parentheses indicate the number of changes between haplotypes.

An analysis of molecular variance was performed using Arlequin 3.5.1.2 [43] and GenAlEx 6.5 [44,45]. A haplotype network (Fig 4) was constructed using DnaSP [46] and PopART 1.7 [47,48].

## Results

The Bayesian tree (Fig 2) and the GARLI consensus tree (Fig 3), along with the haplotype network (Fig 4), suggest a global panmictic population with a high incidence of gene flow among populations from different geographic regions. The presence of polytomies in both trees indicate that there is not enough genetic signal in the CO1 gene to define populations based on their geography alone in this case. The most prominent instance of regional clustering was exhibited by a small North American clade, present with >50% support in both trees, containing only three individuals: U.S.A. 1, U.S.A. 2, and Canada 2. Interestingly, the second Canadian sample (Canada 1) was most closely related to one of the India samples (India 3) in each tree with >50% clade support in both cases. This observation, along with the other India samples being grouped with individuals from Japan and Guyana, suggest that there is gene flow occurring among these regions.

Both trees showed individuals collected from Guyana grouped not only with other Guyana samples, but with samples from all other regions in polytomies, small clades, and weakly supported larger clades. One small Guyanese only clade was present in both trees, with >70% support in each, and contained individuals Guyana 8 and Guyana 18. These two individuals were found in the same area as one another but were collected one year apart. Samples Guyana 8 and Guyana 18 were also grouped together as a distinct haplotype in the haplotype network (Fig 4), lending further support to this clade. The other Guyana only clade to appear in both trees, consisting of individuals Guyana 12 and Guyana 21, fell below 50% support in both trees. One Guyanese sample, Guyana 16, exhibited a distinct haplotype with 54.9% bootstrap support in the GARLI consensus tree, but was grouped with other Guyanese, Japanese, and Indian samples in a large polytomy in the Bayesian tree. The haplotype network did not distinguish Guyana 16 as having a distinct haplotype in comparison to other individuals.

Similar to the Guyanese distribution mentioned above, the distribution of Japan samples in both trees showed them being grouped not only with other Japan samples, but with a mix of individuals from other regions as well. There were two small Japan only clades that appeared in both trees. The first contained individuals Japan 1 and Japan 6, collected from neighboring prefectures in Japan one year apart from one another. The second Japanese only clade contained individuals Japan 5 and Japan 16. However, both of these clades fell shy of having >50% bootstrap support in the GARLI consensus tree.

The only clade not yet mentioned to appear in both trees with >50% support contained individuals Guyana 19 and Japan 3. This clade, along with the distribution of all other individuals in both trees (Figs 2 and 3) and the haplotype network (Fig 4), strongly suggest that gene flow is occurring on a worldwide scale among multiple populations.

Molecular variance analysis in Arlequin returned an Fst value of 0.0426 with 95.74% of variation occurring within populations and 4.26% of variation occurring among populations. A second molecular variance analysis in GenAlEx resulted in a PhiPT value of 0.000 with 100% of variation occurring within populations and 0% occurring among populations. Both of these analyses further imply panmixia and high incidence of gene flow.

The samples India 4 and Korea 1, both sourced from NCBI GenBank, stood out as the most distinct haplotypes in every analysis. It is unclear whether the Korea 1 sample is as distinct as it appears, or if the overall quality of the sequence was not as high as the other sequences used in the analysis. The India 4 sample was listed as “cytochrome oxidase subunit 1-like” on NCBI GenBank, which likely accounts for it being far more distinct than the other CO1 sequences.

## Discussion

Considering the migratory capabilities and extensive ranges of *Pantala flavescens*, that this species may exist as a global panmictic population has been considered possible by researchers over several decades [7,14,23]; yet this theory has not been adequately investigated thus far, and we have little genetic evidence for members of this genus. Our current study may present the first significant evidence to suggest that *P*. *flavescens* should be considered a predominately global panmictic population rather than a series of geographically isolated, distinct populations. Each of our analyses suggest that, given the mitochondrial data collected, gene flow is occurring on a global scale among *P*. *flavescens* populations from various geographic regions, suggesting panmixia. The remarkable, large scale migrations of *P*. *flavescens* are likely the primary contributing factor to the observed high rates of gene flow and diminished genetic diversity.

Previous studies have shown similar high rates of gene flow in *P*. *flavescens*, but on smaller spatial scales. Using randomly amplified polymorphic DNA, Christudhas and Mathai [49] examined genetic diversity among five geographically isolated populations of *P*. *flavescens* within India. Their analysis suggested low genetic diversity and uncovered a high rate of gene flow, which implies panmixia among populations within India where annual migrations are known to occur [50]. The samples from India in our analysis ranged from geographically distant southern (India 2), eastern (India 1), and western (India 3) India (Fig 1); our analyses support the findings presented by Christudhas and Mathai [49], and suggest that the gene flow is occurring on a large scale, spanning continents. Although more data is needed, specifically to expand sampling in Africa and add genetic loci, these two studies suggest that the migrations of *P*. *flavescens* have led there to be significant gene flow and reduced genetic diversity.

Based on what we know of the species, it is likely that *P*. *flavescens* can be considered an obligate migrant [15]. Utilizing adaptations such as an enlarged hind wing base [8,14,15], which is ideal for gliding while expending minimal amounts of energy, *P*. *flavescens* can readily take advantage of prevailing, seasonal winds associated with fronts such as the Intertropical Convergence Zone [6,14,15,23], allowing them to cover extraordinary distances. The ITCZ provides not only the winds necessary to assist in migrations, but the associated rains produce ephemeral freshwater pools that the dragonflies require for reproduction [9,14]. A significant investment in migratory capabilities allow *P*. *flavescens* to follow favorable breeding weather conditions, resulting in the increased potential to reproduce throughout much of the year [16]. Perhaps the most well documented migration of *P*. *flavescens* thus far has been the transoceanic migratory circuit from India to Africa (Fig 1) and back again, influenced by the seasonal shifting of the ITCZ in the region [6,14,23]. Isotopic evidence suggests that the multigenerational journey may total over 18,000km with single individuals traveling over 6,000km during the transoceanic trek from northern India to east Africa [6]. This migration exemplifies how the long distance dispersal capabilities of *P*. *flavescens* are largely passive; a key element in explaining the prevalence of global gene flow in our findings.

Documented observations of *P*. *flavescens* migrating in accordance with seasonal winds in other parts of the world, such as China [9], further reinforce the long distance, passive dispersal capabilities which allow them to circumnavigating the globe. While their reliance on strong winds allows them to cover distances far greater than any other known migratory insect [5,6], these same winds can also be responsible for carrying them to areas far from their normal migratory range [7,15]. With a tendency to migrate in large swarms [5,14], if even a portion of a migratory aggregation were to be consistently carried by winds in a new direction the impact this would have on increasing gene flow between geographic regions is likely to be significant. High rates of gene flow will counteract divergence [51], reducing genetic diversity on a large scale while maintaining a panmictic population.

Passive dispersal has undoubtedly contributed to the observed high rate of gene flow, but it may also be the factor that has resulted in the only documented population of *P*. *flavescens* that represents an exception to the characteristic migration of the species. This population, described by Samways and Osborn [7], is found on Easter Island in the Southeastern Pacific Ocean and has developed both behavioral and morphological characteristics that indicate they are non-migratory. In addition to being non-natives, they are the only species of dragonfly found on Easter Island and most likely arrived at this remote location as a result of wind-assisted passive dispersal. While some oceanic islands serve as stopping points along a migratory route, such as the case with the Maldives [14], this non-migratory Easter Island population raises two important questions: how many other populations exist in extreme isolation and can they still be considered part of a global panmictic population? Further genetic analysis of the Easter Island population, as well as any others that may exist, will be required to answer this question definitively. As for now it appears that extreme isolation may be the one factor that can influence divergence in the species as no other geographic features have proven to be a challenge up to this point.

The only individuals that exhibited a distinct haplotype in our study were the India 4 and Korea 1 samples. The degree to which the India 4 sequence differed from the rest of the sequences is likely explained by it being "similar to cytochrome oxidase subunit 1" whereas all other sequences were strictly CO1. Regarding the Korea 1 sequence, as it was sourced from NCBI GenBank, we are unable to speak to the quality of the sample or sequence in this case. Based on the Bayesian tree in Fig 2, Korea 1 could indicate possible divergence and evolution of new haplotype in the region. However, support for this is not as strong in the GARLI tree in Fig 3. Considering the relation of the second Korean sample (Korea 2) to the other individuals in both trees (Figs 2 and 3) and the haplotype network (Fig 4), it would seem unlikely that Korea 1 is as unique as it may appear. However, the possibility that it is a distinct haplotype must be considered and cannot be easily dismissed given the available data. Precise data regarding the collection location and date was not available at the time of writing for this sample. An in depth analysis utilizing said data, in comparison with any known migratory populations in the region, could provide further insight into how distinct this sequence may be.

Gene flow is present. As illustrated in our reconstructed phylogenetic trees (Figs 2 and 3), haplotype network (Fig 4), and molecular variance analyses, gene flow is occurring on a global scale among geographic regions. The overall lack of regionally discernible genetic structure in our samples may provide valuable insight regarding the consistency and stability of the migratory routes of *P*. *flavescens*. Strict adherence to regional migration routes, expansive as they may be, would likely result in a relatively small number of very large populations spread across the globe [15]. If this concept held true we would have expected to see distinct regional clades in our results; instead we find a mix of polytomies and small clades consisting of both Asian and New World individuals.

The North American clade present in both trees (Figs 2 and 3) containing all individuals from Texas, U.S.A. (U.S.A. 1, U.S.A. 2) and one individual from New Brunswick, Canada (Canada 2) is the primary exception. However, the consistent grouping of the second sample from New Brunswick, Canada (Canada 1) with the Indian sample from Gujarat, India (India 3) suggests that although there may be a North American migration route, North American individuals are breeding with individuals from other continents. Little research has been dedicated to uncovering North American migratory circuits of *P*. *flavescens* but, based on the aforementioned clade in our study, it is a topic worthy of further exploration. Future work should sample *P*. *flavescens* from across Canada, the United States, and Mexico, and evaluate how much true gene flow there is between North America and other continents. Indeed, there could be overlapping, smaller migratory routes that connect North American populations to South American populations, with there being isolation by distance. We do not see this here, but that is likely due to our very small number of US and Canadian samples. In addition, an emphasis on connections to West Africa would be interesting to explore, as hurricanes may bring *P*. *flavescens* there; hurricanes have been shown to bring the African dragonfly *Anax ephippiger* to the West Indies [52].

Significant support for dedicated South American and Asian clades was lacking in our analyses. The small Guyanese clade, containing individuals Guyana 8 and Guyana 18, and the comparably sized Japanese clade, containing individuals Japan 5 and Japan 16, were the only exceptions with adequate support in both trees (Figs 2 and 3). These clades imply that at least some degree of structure may exist in these regions, but the majority of samples from both these countries were consistently grouped with all other regions in the analysis. The significantly supported Japan-Guyana mixed clade, containing individuals Guyana 19 and Japan 3, exemplify that while some degree of structure may exist within these regions there is still a significant amount of gene flow occurring between them; a trend that holds true for all regions analyzed in this study.

A larger data set of *P*. *flavescen*s samples collected from an even wider geographic range would be the first step in further assessing rates of gene flow and fluctuations in genetic diversity. In addition to increasing the volume of samples, strategic collection at specific locations and dates coinciding with migratory routes may provide valuable information regarding the stability of these migrations. Techniques such as attaching radio transmitters to migrating individuals have been used to study other migratory dragonflies [12], but considering the scale of *Pantala* migrations it is unlikely to be effective solution for a multitude of reasons. As any direct measurements of dispersal will encompass a significant number of challenges, genetic analyses used in this study, and isotope analyses used by Hobson et al. [6], will likely prove to be the most efficient method for studying the migrations and population structure of *P*. *flavescens*.

Although the use of the mitochondrial CO1 gene has proven to be an effective marker for studying divergence within and among species [53], further genetic analyses would help to evaluate any findings uncovered thus far. The rate of evolution exhibited by mitochondrial DNA is quicker than that of nuclear DNA, making it unlikely that additional analyses using nuclear genes in place of mitochondrial genes would be more successful in uncovering any recently developed population structure in the species. It is still possible that further genetic analyses using rapidly evolving genetic markers, such as additional mitochondrial genes and microsatellites, may uncover existing structure within populations that the use of CO1 alone was not able to accurately predict. Previous studies on the age of Odonata, which included *Pantala*, support an origin of the species in the mid to early Miocene [54], yet information regarding the biogeographical origins of the species remain unresolved. Genetic analyses with more loci over an even wider geographic range will be required to address this question.

Our results strongly suggest that *P*. *flavescens* exists in a large panmictic population and that there is worldwide gene flow among populations. However, there is still a significant amount of work to be done in terms of studying *Pantala* on a global scale. In addition to examining their population structure, additional studies regarding the potential ecological impacts of their migratory behavior are worthy of further discussion. As for the future of the species, questions concerning the impact that climate change may have on their migrations and range expansion are noteworthy considerations. Importantly, our study is the first to suggest that genes are being shared among individuals across the globe; this lays the groundwork for future studies, but for this extensive taxon sampling needs to be undertaken. Maintaining a global perspective in future studies of *P*. *flavescens* will prove to be essential in furthering our understanding of these extraordinary “wandering gliders”.
